# Design, Testing, and Validation of a Soft Robotic Sensor Array Integrated with Flexible Electronics for Mapping Cardiac Arrhythmias

**DOI:** 10.3390/mi15111393

**Published:** 2024-11-18

**Authors:** Abdellatif Ait Lahcen, Michael Labib, Alexandre Caprio, Mohsen Annabestani, Lina Sanchez-Botero, Weihow Hsue, Christopher F. Liu, Simon Dunham, Bobak Mosadegh

**Affiliations:** 1 Dalio Institute of Cardiovascular Imaging, Department of Radiology, Weill Cornell Medicine, New York, NY 10021, USA; 2 Department of Clinical Sciences, College of Veterinary Medicine, Cornell University, Ithaca, NY 14853, USA; 3 Department of Cardiology, Weill Cornell Medicine, New York, NY 10021, USA

**Keywords:** cardiac mapping, soft robotic sensor arrays, catheter, flexible printed circuit board (PCB), minimally invasive procedure

## Abstract

Cardiac mapping is a crucial procedure for diagnosing and treating cardiac arrhythmias. Still, current clinical techniques face limitations including insufficient electrode coverage, poor conformability to complex heart chamber geometries, and high costs. This study explores the design, testing, and validation of a 64-electrode soft robotic catheter that addresses these challenges in cardiac mapping. A dual-layer flexible printed circuit board (PCB) was designed and integrated with sensors into a soft robotic sensor array (SRSA) assembly. Design considerations included flex PCB layout, routing, integration, conformity to heart chambers, sensor placement, and catheter durability. Rigorous SRSA in vitro testing evaluated the burst/leakage pressure, block force for electrode contact, mechanical integrity, and environmental resilience. For in vivo validation, a porcine model was used to demonstrate the successful deployment, conformability, and acquisition of electrograms in both the ventricles and atria. This catheter-deployable SRSA represents a meaningful step towards translating the integration of soft robotic actuators and stretchable electronics for clinical use, showcasing the unique mechanical and electrical performance that these designs enable. The high-density electrode array enabled rapid 2 s data acquisition with detailed spatial and temporal resolution, as illustrated by the clear and consistent cardiac signals recorded across all electrodes. The future of this work will lie in enabling high-density, anatomically conformable devices for detailed cardiac mapping to guide ablation therapy and other interventions.

## 1. Introduction

Cardiac arrhythmias are highly prevalent conditions; atrial fibrillation (AFib) currently affects over 37 million people globally and over 6 million in the United States alone [1]. By 2030, it is estimated that cases of AFib in the United States will rise to 12.1 million [2]. Other arrhythmias, including atrial flutter, premature ventricular contractions, and ventricular tachycardia, also afflict millions [3]. Accurate mapping of cardiac electrical activity is crucial for diagnosing and guiding ablation therapy for these conditions. However, the current generation of cardiac mapping technologies is constrained by insufficient electrode coverage, inability to conform to complex heart chamber geometries, and high costs [4].

A primary challenge in cardiac mapping is the insufficient electrode coverage from current mapping devices. When these devices are moved into contact with the cardiac surface, the complex geometries of real heart chambers mean that only a portion of the electrodes establish contact with tissue [5]. Some small devices do not suffer from this challenge but only provide signals from a small area of the heart chamber. As such, the proportion of the chamber where spatiotemporally resolved (i.e., the number of acquired electrograms in a cardiac map that are associated with a given arrhythmia or subsequent cardiac cycles) electrical signals can be acquired is limited. This challenge is inherent to the physical hardware of minimally invasive diagnostic catheters that have one, or a few, sensors at their tip, or are composed of an array of electrodes that have a relatively small area compared to a cardiac chamber [6]. Additionally, achieving optimal conformability of the catheters to the cardiac chamber walls remains an ongoing challenge [7]. Furthermore, some mapping techniques may not allow for the simultaneous acquisition of electrical signals from multiple sites, hindering the creation of detailed and comprehensive maps of cardiac electrical activity [8]. Furthermore, the relative rigidity and inflexibility of cardiac mapping devices have historically limited their ability to conform to the heart’s surface and accommodate the deformations associated with cardiac motion [7]. This leads to poor detection of AFib drivers due to their sequential spatiotemporal characteristics, intermittent firing, and intricate atrial anatomy. Ultimately, these factors affect the accuracy and quality of the acquired electrical signals [9]. As a result of poor electrode positioning and tissue contact, less than 50% of the sensors provide meaningful data [5,10]. Moreover, existing clinical cardiac mapping technologies can be expensive to develop [9,11]. This inevitably limits their widespread adoption and accessibility and serves as a challenge for scalability.

Recent advances in soft robotics offer promising solutions to overcome the challenges of traditional mapping catheters. Soft robotic systems leverage flexible and stretchable materials to better conform to dynamic surfaces, without the need for complex feedback and control systems [6]. The integration of soft robotics and stretchable electronics has shown great promise in improving the conformability of cardiac mapping devices to the complex anatomy of the heart. In this context, Chen et al. developed a soft, bioresorbable, transparent microelectrode array platform that can provide organ-conformal cardiac interfacing for clinically relevant spatiotemporal mapping [12]. However, this approach is limited by the need for open-heart surgery and the inability to map the inside of heart chambers (i.e., the endocardium). Farokhnia et al. developed an inflatable basket design that allows a soft robotic device to be deployed through a catheter to enhance conformability to the heart's left atrium [13]. The device comprises a soft elastomeric balloon with an embedded stretchable sensor and actuator network that enables multimodal mapping and modulation. A CO_2_ laser was used to design and fabricate these soft robotic actuators, and this proved to be a key advancement in the cost-effective fabrication of cardiac catheters. Ait Lahcen et al. presented a novel method for creating stretchable electronics from dual-layer flex PCB to serve as a soft robotic sensor array (SRSA) platform for cardiac voltage mapping applications [14]. Inspired by Kashyap et al., laser-based post-processing methods were used to convert inelastic flex PCBs into stretchable electrode arrays [6]. When used with serpentine electrical traces, this approach can yield stretchable electronic configurations that can accommodate significant strain. In vitro and in vivo testing showed the versatility of the SRSA in acquiring electrocardiograms in various environments.

These advancements in soft robotics, stretchable electronics, and laser-based fabrication techniques have enabled the development of highly conformable cardiac mapping devices that can adapt to the intricate geometries of the heart. For example, Han et al. developed flexible and stretchable electronics that tightly integrate with the epicardial surface for high-density mapping [15]. Prior research on minimally invasive sensor deployment has explored various strategies to integrate multifunctional soft sensor arrays with conventional catheters and inflatable medical balloons for cardiac applications. Han et al. made significant advancements in this field by developing catheter-integrated soft multilayer electronic arrays for multiplexed sensing, deployed on an actuatable balloon during cardiac surgery [15]. Their device features a multilayer design with separate layers for electrodes (64 electrodes in an 8 × 8 array), temperature sensors, and pressure sensors. The device is fully integrated with commercial and customized catheters, allowing minimally invasive deployment. Its key capabilities include high-density multimodal mapping, conformal contact with tissue surfaces, and the simultaneous multimodal operation of sensors and actuators. Han et al. validated their device’s efficacy in sensing, actuation, and conformability to tissue surfaces by testing their catheter systems in New Zealand white rabbits, Langendorff rabbit heart models, and donor human hearts. Through in vitro performance and reliability tests, the device’s safety was assessed to evaluate biocompatibility and tissue conformability without damage. The work by Han et al. represents a significant advancement in the field of minimally invasive sensor deployment by showcasing the potential of soft multilayer electronic arrays that are catheter-integrated for multiplexed sensing and actuation during cardiac surgery.

As shown in Table 1 above, our study shows promise for clinical translation in comparison to the literature. Although Han et al. [15] and Kashyap et al. [6] used a device with a higher electrode count, our device demonstrates sufficient electrode coverage (64), in vivo functionality, and a simplified fabrication process using medical-grade materials. Our integration of soft robotics and flex PCBs to enhance cardiac mapping in a minimally invasive form has been validated through comprehensive testing. Ranking alongside previous publications, our study contributes to the innovation necessary for the clinical translation of cardiac mapping technology.

This work aims to develop and validate an innovative integrated SRSA for enhanced cardiac mapping. By incorporating four flexible 16-electrode arrays into 4-spline soft robotic actuators, an SRSA with a total of 64 electrodes was integrated onto a minimally invasive catheter. Our approach addresses the limitations of traditional methods, such as insufficient electrode coverage and poor conformability to cardiac anatomy, by designing a whole chamber device that mimics traditional basket catheters but replaces nitinol with soft robotic actuators. This design ensures better contact with cardiac tissue, improving the accuracy and resolution of electrical maps. The novelty of this work lies in its unique integration of soft robotics and stretchable electronics to allow for the effective deployment of electrode arrays via a minimally invasive catheter. We conducted extensive benchtop tests to evaluate the device’s mechanical integrity, demonstrating its durability and robustness. Validation of the device’s performance was done in vivo using a porcine model, which demonstrates the potential for spatiotemporally resolved electrocardiograms to be acquired. This research provides promise for a clinically translational SRSA that enhances the efficiency and effectiveness of cardiac mapping, guiding more precise and targeted arrhythmia treatments. Our findings represent a transformative step forward in cardiac electrophysiology, paving the way for more accurate and less invasive diagnostic tools in clinical practice by using soft robotics.

## 2. Experimental Methods

### 2.1. SRSA Device Assembly Protocol

#### 2.1.1. Flex PCB Sensor Design and Flex PCB Extender Design

The flex PCB and their extenders used in our study were designed using Rhinoceros 6 (Rhino 6, North Seattle, WA, USA). The designed inelastic flex PCBs and extenders were sourced from a conventional flex-PCB vendor (EPEC Engineered Technologies, New Bedford, MA, USA). The design was based on multiple iterations of previous research published by our group and addressed several limitations that we encountered previously, such as the oxidation of the copper electrodes, which were addressed by using an Electroless Nickel Immersion Gold (ENIG) finish. Additionally, this new design allows a dual-layer configuration of the electrodes at the flex PCB level. The laser-based post-processing technique is used for the conversion process of the inelastic flex PCBs to stretchable electrode arrays. For additional technical depth, we have referenced our recent work [14], which provides extensive documentation of the parameters, optimization process, and technical limitations of this technique. However, several critical challenges need to be addressed for practical applications. Key limitations of the laser post-processing technique include concerns about mechanical durability and delamination under repeated deformation, material biocompatibility, signal stability, and manufacturing scalability. These challenges necessitate comprehensive validation studies before clinical implementation.

#### 2.1.2. The Four-Legged SRSA Fabrication Method

Two layers of thermoplastic polyurethane (TPU) (purchased from ACP Composites, Livermore, CA, USA) were heat pressed as a single sheet at 137 °C for 10 min and then allowed to cool for 2 min. Our motivation for the use of TPU as a material was its excellent properties of flexibility and thermal/electrical isolation. Then, the edges were cut off and it was divided into enough parts to cover the actuator. A strip of Polytetrafluoroethylene (PTFE) material (purchased from McMaster Carr, Robbinsville Twp, NJ, USA) was used as an inlet that was sandwiched between two layers of TPU (two layers on the top and two layers on the bottom) and was heat pressed at 137 °C for 10 min, then left to cool down on the tray for ~2 min. A square of polyvinyl alcohol (PVA, purchased from JOANN, Colonia, NJ, USA) film was cut and heat pressed to perfectly flatten it at 137 °C for 10 min and then was immediately taken out on a flat surface. The PVA was cut into the same design as the inner actuator using a CO_2_ laser cutter (VL300 Universal laser systems, 30 W—600 × 300 mm), Universal laser systems, Scottsdale, AZ, USA. The PVA was heat pressed on one of the two layers of TPU at 137 °C for 10 min and was left to cool down on the tray for about 2 min. A hole was punctured in the middle of the other two layers of TPU (4 mm diameter biopsy punch). The edges of the inlet were cut with a border of about 1 mm, but all edges were removed for the circular part. The tab was folded and was held still with a clamp.

The PTFE inlet and the two-layer TPU were combined via the punctured hole, a PTFE film was used to prevent the inlet binding with the two-layer TPU, and the assembly was heat pressed at 137 °C for 10 min, then was left to cool down on the tray for about 2 min. The extra PTFE was cut and folded on itself. The two layers of the TPU/actuator and the two layers of the TPU/PTFE inlet were combined and heat pressed at 152 °C for 10 min then left to cool down on the tray for about 5 min. The edges of the actuator were cut off with a border of about 1 mm, and the extremity of the balloon was cut off with edges of about 10 mm.

The windows were laser cut from one layer of the TPU. The windows were heat pressed onto the flex PCB at 137 °C for 10 min. The edges of the GPU windows/flex PCBs were cut up to the wires. The windows/flex PCB was heat pressed onto each leg of the SRSA at 137 °C for 10 min and was left to cool down on the tray for about 2 min. The sheet of PTFE was removed from the SRSA, the edge of the inlet was cut, and the PTFE within it was also removed. To ensure successful fabrication, the SRSA was perfused with water, and each leg was massaged gently until all the PVA sacrificial layer was dissolved and removed. The inner rod was inserted inside the SRSA’s inlet, was glued together, and was wrapped with PTFE tape. Each leg was attached to the outer rod and heat shrink tubing and glue were used to hold it together.

**Figure 1:**
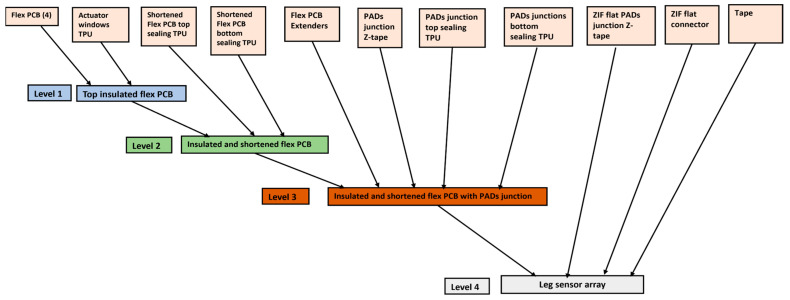
Schematic illustration of the four-legged sensor array assembly.

As indicated in Figure 1**,** The four-legged sensor array was fabricated using flex PCBs, TPU components for sealing and actuation windows, PCB extenders, PADs junctions with Z-tape attachments, and tape connectors, all assembled in a four-level hierarchical structure.

#### 2.1.3. Catheter Positioning and Four-Legged SRSA Access

The four-legged SRSA is designed to be deployed via an articulating Destino Twist 6.5 Fr catheter (purchased from Oscor, Palm Harbor, FL, USA) allowing efficient deployment and articulation. The device is mounted on the catheter with a telescoping 6.5 Fr introducer that served as the inflation lumen such that the device was connected to the distal end of the introducer, where the PTFE inlet described above was attached. This delivery catheter can be pushed through a 13.8 Fr Destino Twist guiding catheter for deployment. Once deployed, the inner catheter can be used to rotate the SRSA, inflate/deflate it, and adjust the shape of the device by changing the distance between the distal and proximal ends of the device using the telescoping introducer. The best positioning of the SRSA is of crucial importance to ensure the best deployment of the device later inside the cardiac chamber.

**Figure 2:**
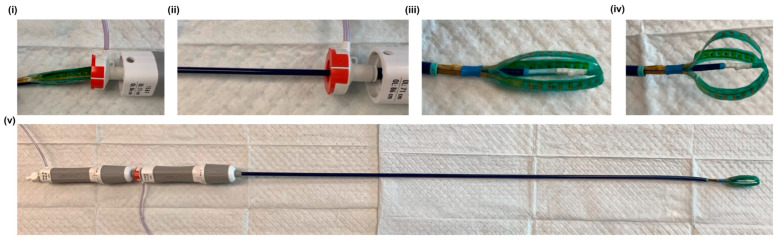
The SRSA device’s integration steps start with (**i**) SRSA distal hub insertion into the Oscor catheter inlet, (**ii**) the insertion of the 6.5 Fr inner catheter into the outer 13.8 Fr catheter, (**iii**) the successful insertion of the four-legged SRSA into the catheter, (**iv**) the deployment of the four-legged SRSA, and (**v**) the whole device showing the four-legged SRSA integrated with the catheter.

The step-by-step process of inserting the delivery catheter through the guiding catheter is shown in Figure 2. It begins with the insertion of the SRSA distal hub into the Oscor guiding catheter hemostatic valves (Figure 2i), followed by the careful feeding of the 6.5 Fr introducer, creating a coaxial system (Figure 2ii). The third image displays the successful insertion of the deflated four-legged SRSA through the guiding catheter lumen (Figure 2iii). The fourth image depicts the deployment of the four-legged SRSA outside the catheter tip (Figure 2iv). The final image presents the fully assembled device, demonstrating the entire length of the catheter with the integrated SRSA.

### 2.2. Rigid PCB Design and Integration with NI Data Acquisition

#### 2.2.1. Custom-Made Rigid PCB Design

**Figure 3:**
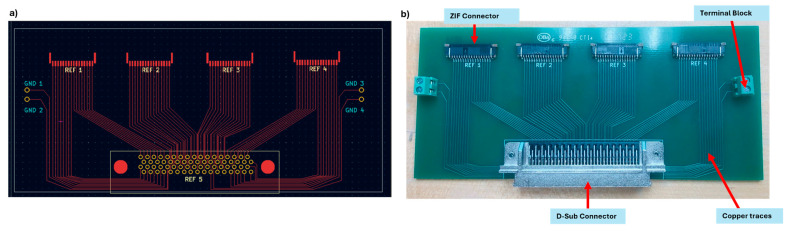
Custom-made rigid PCB board. (**a**) The KiCAD design of the rigid PCB. (**b**) The custom-made rigid PCB used to connect the SRSA with the National Instruments readout.

The custom rigid PCB was designed using the KiCAD 6.0 software, tailored specifically for data acquisition in our research. As shown in Figure 3, this compact PCB features a rigid substrate that integrates four Zero Insertion Force (ZIF) connectors, allowing easy and secure connections to external devices. Additionally, the board includes a D-sub connector for interfacing with the National Instruments Data Acquisition (NI DAQ USB-6225, Austin, TX, USA) Board and four ground pins connected by two terminal blocks. Assembled by Onboard Circuits (Scottland, AZ, USA), this rigid PCB seamlessly integrates data acquisition capabilities, enhancing reliability and functionality in our experiments.

#### 2.2.2. Integration of the SRSA Device with the Data Acquisitor

The SRSA device was integrated with the NI DAQ board thanks to the custom-made rigid PCB. Four ZIF connectors, each containing 16 pins, were used to connect each leg of the SRSA. The DAQ serves as a readout tool for the electrical signals acquired by the SRSA device.

#### 2.2.3. LabView Program Custom-Interface

A custom-made interface was made using LabView 2022 Q3 to collect, save, and visualize data in real-time. The interface allows for the simultaneous acquisition of electrical signals from all 64 electrodes. The program allows the selection of specific electrodes, setting of thresholds, and application of filters.

### 2.3. Bench Testing

#### Validation Tests

- Burst/leakage testing

To assess the pressure integrity of the actuator sub-assembly and to establish the maximum pressure thresholds, burst/leakage tests were performed. We connected the assembly to an inflation/deflation pressure pump, the NE-300 Syringe Pump (New Era Pump Systems, Farmingdale, NY, USA), and gradually increased the internal pressure while monitoring it with a pressure transducer with 0 to 100 Psi (purchased from Transducers, Cincinnati, OH, USA). We inflated the device until we reached the predetermined limit or until failure occurred. Throughout the test, we closely observed the actuator for any signs of deformation, leakage, or stress. We documented the specific pressure levels at which the actuator experienced structural failure and when initial leakage began. A custom-made Arduino Uno was used for the data acquisition of the pressure levels and a software called CoolTerm version 2.1.0 was used to analyze the data.

b. Actuator’s block force measurements

Block force measurements were performed to assess the actuator’s functionality. These measurements were performed using a 3D printed jig, which served as a support to hold the SRSA spline in a specific position relative to the weighing balance (purchased from OHAUS, Parsippany, NJ, USA).

c. Durability tests

To evaluate the long-term reliability of the prepared actuators, durability testing was conducted on multiple samples. Each actuator underwent 100 actuation cycles, simulating extended periods of use. Block force measurements were performed as described in the Actuator’s block force measurements Section before and after cycling. A digital multimeter (purchased from SparkFun Electronics, Boulder, CO, USA) was used to take the electrical measurements before and after this endurance test to assess electrical performance stability. The radius of curvature after a certain number of actuation cycles was calculated using Kinovea-0.9.5 software version.

d. Environmental testing

To assess the actuator’s durability in physiological conditions, comprehensive environmental tests were conducted. Baseline electrical resistance between neighboring and cross electrodes was initially recorded, and the initial condition of the TPU windows was documented. The measurements of electrical resistance were performed using a digital multimeter from SparkFun Electronics. The actuator was then immersed in a body-mimicking solution within a sterile container. After 24 h, the actuator was removed, resistance measurements were repeated, TPU windows were inspected, and electrode functionality was verified before the actuator was returned to the solution. This process was repeated after one week of immersion. Throughout the test, any changes in electrical resistance, TPU window integrity, and overall functionality were monitored. Standard actuator function tests were performed post-immersion and compared to the pre-immersion baseline. The data were analyzed to evaluate changes over time, assessing electrical resistance variations, physical alterations, and electrode performance.

e. Junction Integrity Evaluation

To ensure the reliability of our device, we conducted comprehensive mechanical assessments focusing on the critical junction between the flexible PCB pads and extender pads. This area is particularly susceptible to stress and potential failure during operation. Tensile tests were conducted (Instron universal testing system) to quantify the junction’s strength and identify its failure thresholds. Tensile test characteristics were evaluated and synchronized with simultaneous video recording and electrical measurements of the junction. This allowed us to determine the exact moment and location of any mechanical failures, providing insights into the mechanisms of failure.

f. Medical-grade polyurethane tests

To validate the SRSA device, we conducted an extensive study using various medical-grade polyurethanes that have a track record of use in FDA-approved devices. The experiment focused on a range of polyurethane-based materials, including polyether block amide (Pebax 5533 SA01 Med; Pebax 7233 SA01 Med), the thermoplastic styrene-based polymer known as TSP (1031-3400 70A; 1051-5800; 1065-4500), and Pellethane (2363 80AE; 90AE), each tested at different widths (Polyzen; Apex, NC, USA). Multiple actuators were fabricated using these materials to determine the optimal choice for our device. The validation process involved block force measurement tests as described above, which were designed to assess the performance of the device under diverse conditions. This comprehensive approach allowed us to evaluate the suitability and reliability of each material for the SRSA application.

### 2.4. In Vivo Validation of the Device in Animal Models

The in vivo validation of the SRSA device was conducted over two days at the animal facility of the Memorial Sloan Kettering Cancer Center (MSKCC) in New York City. The experiments utilized both two-legged and four-legged SRSA configurations of the device to assess its performance in various cardiac chambers of porcine models. Under fluoroscopic, transesophageal echocardiographic (TTE), and intracardiac echocardiographic (ICE) guidance (Philips VeriSight, San Diego, CA, USA), the devices were sequentially deployed into the right atrium, right ventricle, left atrium, and left ventricle. The procedure involved a transseptal puncture to access the left side of the heart and the use of iodinated contrast agents to enhance visibility. Throughout the experiment, our research team carefully monitored device positioning, wall contact, and signal quality. We made real-time adjustments to optimize device placement and performance, capturing both fluoroscopic and echocardiographic images at key stages.

## 3. Results and Discussion

### 3.1. SRSA Device Overview

**Figure 4:**
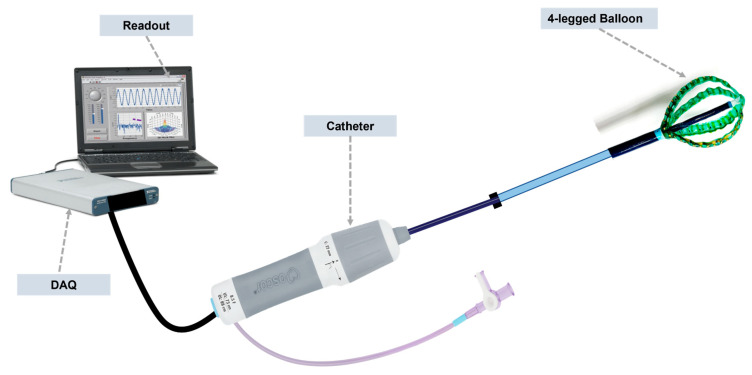
Soft robotic sensor array assembly that shows the final catheter-delivered design.

The SRSA device demonstrates a significant advancement in catheter-delivered biomedical technology. Figure 4 illustrates the device’s final design, which integrates multiple components into a compact, functional unit suitable for minimally invasive procedures. The development process of this device emphasizes the critical importance of meeting the Food and Drug Administration (FDA) requirements for medical device translation. Care was taken to ensure that all device components were developed from materials that have predicate use in devices with similar clinical uses (particularly cardiac mapping devices). To understand the test requirements, a comprehensive device component and assembly list was developed.

All device components were documented, categorizing them into three distinct levels: individual components, minor sub-assemblies, and major device assemblies (Figure S1). This hierarchical organization, detailed in the Supplementary Materials (Tables S1–S3), proved invaluable in structuring the testing process. The function of these components and their associated risks motivated the testing described below. These tests encompass various aspects of the device’s functionality, safety, and reliability.

The developed SRSA cardiac catheter device was meticulously engineered and validated according to medical industry standards, incorporating comprehensive testing across multiple critical parameters. The design incorporated medical-grade TPU materials and ENIG-coated flex PCBs, with proven durability through rigorous mechanical testing that demonstrated strong junction strength and pressure tolerance. Environmental testing confirmed device stability in physiological conditions over 7 days, with maintained functionality in saline environments and across relevant temperature ranges. The device was designed as a single-use instrument capable of 4–8 h procedure durations, with verified sterilization compatibility and an intended 1-year shelf-life, pending validation. All testing protocols exceeded typical procedural requirements with appropriate safety factors, though additional validation studies including accelerated aging and environmental exposure are planned for regulatory submission.

The SRSA device uses materials with established blood compatibility and biocompatibility histories in FDA-approved cardiac devices.

Blood-Contacting Components:

- TPU materials sourced from Polyzen with established use in blood-contacting devices.
- ENIG-coated flex PCBs commonly used in cardiac catheters;
- All materials selected have documented ISO 10993-4 [17] compliance.

Biocompatibility Considerations:

- Pellethane and TSP are medical-grade polyurethanes with proven biocompatibility;
- These materials are currently used in commercial cardiac mapping catheters;
- Materials have established ISO 10993-5 [18] cytotoxicity data.

Risk Mitigation:

- Short-term contact duration (<24 h);
- Single-use design eliminates reprocessing risks;
- TPU encapsulation provides an additional barrier.

While formal ISO 10993-4/5 [17,18] testing will be conducted for regulatory submission, the current prototype uses materials with established safety profiles in similar cardiac applications. This approach allows rapid iteration while maintaining safety standards through material selection.

### 3.2. SRSA In Vitro Tests

The SRSA developed for cardiac mapping applications underwent a comprehensive series of in vitro tests to ensure its reliability, safety, and functionality for its eventual use in a clinical setting. These tests encompassed a wide range of performance metrics and environmental factors. Burst and leakage tests were conducted to determine the maximum pressure and volume limits before failure, providing crucial data for safe operational parameters. Block force measurements were performed to quantify the actuator’s strength and capability. Durability was assessed through multiple actuation cycles, simulating long-term use. Environmental tests mimicking cardiac conditions evaluated the actuator’s ability to maintain functionality in physiologically relevant settings. Mechanical integrity tests focused on characterizing the robustness of critical joints and connections. Additionally, the actuator’s compatibility with FDA-approved medical-grade films was examined to ensure its suitability for clinical use. This rigorous testing regimen aimed to determine the actuator’s performance, longevity, and safety for its intended cardiac applications. The section below shows in detail the different tests performed to validate the device.

#### 3.2.1. Burst/Leakage Tests

To assess the pressure integrity of the actuator sub-assembly and establish maximum pressure thresholds, burst/leakage was conducted. The test specifications were designed to exceed physiological cardiac pressures with appropriate safety margins.

Test setup:

- Inflation medium: 0.9% sterile saline solution at 37 °C;
- Pressure pump: NE-300 Syringe Pump;
- Pressure monitoring: calibrated transducer (0–100 PSI, Transducers, Cincinnati, OH, USA);
- Data acquisition: Arduino Uno with CoolTerm software;
- Temperature control: 37 °C water bath.

Testing parameters:

- Working pressure range: 0–186 kPa (0–27 PSI);
- Target burst pressure: >200 kPa (>29 PSI);
- Safety factor: >3× normal physiological pressure;
- Sample size: multiple actuators tested (n = 3 per configuration).

Our testing showed the following:

- Standard actuator burst pressure: 186 kPa;
- Sensing actuator burst pressure: 186 kPa;
- No compromise in structural integrity with integrated sensing;
- Failure modes were properly characterized and documented.

The use of saline solution at a physiological temperature provides realistic testing conditions that match the intended use environment inside cardiac chambers.

The burst and leakage tests conducted on the SRSA actuator, as illustrated in Figure S3, provide an overview of the device’s performance and durability. The figure details the fabrication process, test setup (Figure S3b), and results through multiple components (Figure S3a–i). The experiments were designed to simulate physiological conditions, with actuators immersed in 0.9% saline solution at 37 °C (Figure S3c). Pressure vs volume plots for actuators with and without sensing electrodes reveal burst points at 102.7 kPa and 107.5 kPa, respectively, indicating that the integration of sensing capabilities does not compromise the actuator’s structural integrity.

A key aspect of this study focused on investigating the effect of varying the PVA sacrificial-layer width on actuator performance. As shown in Figure S3i, a correlation was observed between the inner actuator width and burst volume, indicating that adjustments to the sacrificial-layer width could optimize the actuator’s performance. This finding, supported by a comprehensive approach that includes design modifications, physiological simulations, and structural variations, provides valuable insights for improving the reliability and efficacy of the actuator, particularly in cardiac applications. This analysis underscores the potential of these structural adjustments to refine device functionality, reliability, and efficacy in cardiac applications.

Table 2 further enriches the study by summarizing key quantitative and qualitative data for the different tested samples. The actuator sub-assembly with a four-legged extension demonstrated the highest maximum volume (6.89 mL) and volume at burst (12 mL), suggesting an optimal balance of flexibility and durability. Interestingly, samples with varying PVA widths showed different performance characteristics. The 4.85 mm width sample achieved the highest maximum volume (7.29 mL) and a high volume at burst (11.20 mL), while the 3.85 mm width sample reached the highest maximum pressure (241 kPa) but experienced leakage issues.

The failure modes described in Table 2, including elastomer extension, pleat window detachment, and leakage, provide valuable insights into the actuator’s behavior under stress. This information is crucial for identifying weak points in the design and guiding future improvements. Overall, these tests, examining various designs and conditions, establish a solid foundation for optimizing the SRSA actuator’s performance and reliability in cardiac applications. The results underscore the importance of balancing flexibility, strength, and volume capacity in the development of soft robotic actuators for medical use. These tests were selected as part of an internal risk assessment for the simulated use conditions.

#### 3.2.2. Actuator Block Force Measurements Testing

**Figure 5:**
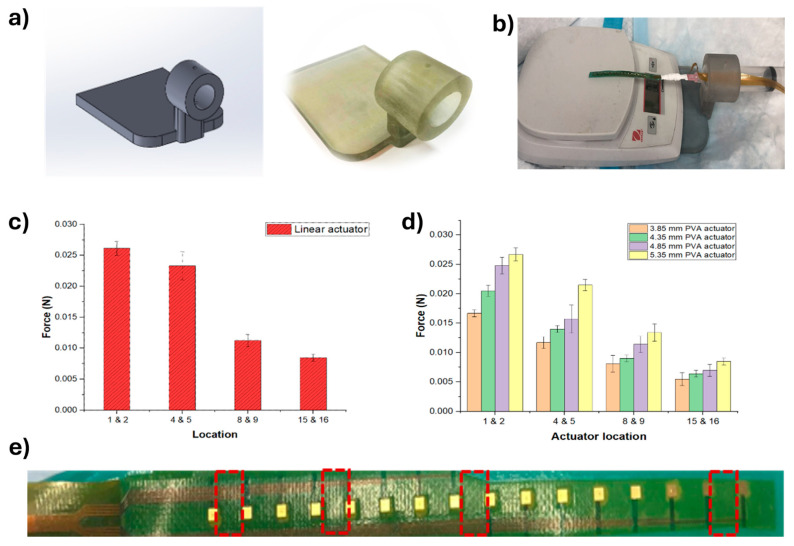
Bench testing performance: (**a**) designed 3D printed mock, (**b**) experiment setup, (**c**) force vs electrode location on the linear actuator, (**d**) force vs actuator location by the width of PVA, and (**e**) linear actuator and location of electrodes shown as red boxes.

The block force measurements are illustrated in Figure 5, which consists of five parts. Figure 5a shows the designed 3D printed part created using SolidWorks 2023 software, including both the model and an actual image of the printed design. This part serves as a support structure to secure the actuator during the block force measurements. Figure 5b displays the experimental setup, featuring the 3D-printed part, the linear actuator, and the force measurement apparatus. Figure 5c presents a bar chart of the force versus electrode location on the linear actuator, illustrating that the force dissipates as the block force measurement progresses from the first and second electrodes to the fifteenth and sixteenth electrodes. Figure 5d shows the force versus actuator location by the width of the PVA, highlighting how the force varies with different actuator locations and the corresponding width of the PVA. Finally, Figure 5e displays the linear actuator and indicates the specific location where the blocking force is applied, providing context for the force application point within the experimental setup.

The results demonstrate that the force exerted by the linear actuator is not uniformly distributed, decreasing as the distance from the initial electrodes increases. This variation in force distribution highlights the importance of precise control and measurement in experiments involving linear actuators. Additionally, the interaction between the actuator and the PVA significantly affects the force measurements, as shown by the variation in force with different actuator locations and PVA widths. The experimental setup, including the use of the 3D-printed part, provides a stable and customizable support structure, enhancing the accuracy and repeatability of the measurements. These findings emphasize the impact of physical parameters on force distribution and application in such experimental setups.

#### 3.2.3. Actuator Durability

**Figure 6:**
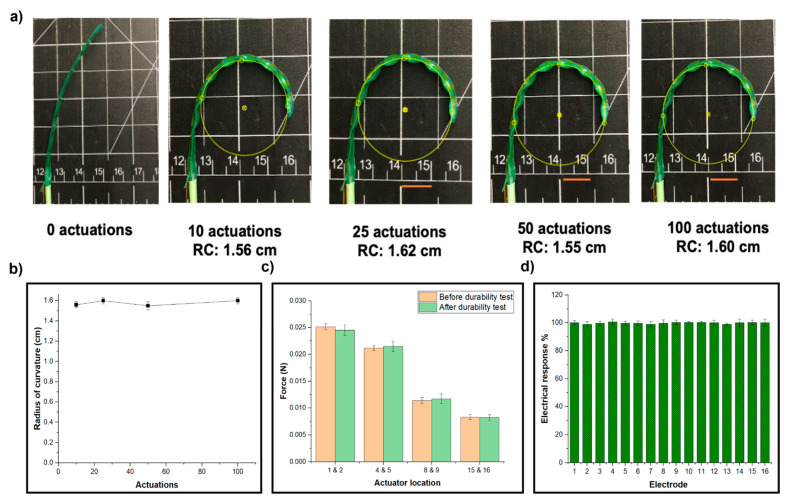
(**a**) An evaluation of conformability for multiple actuations using a deflection analysis (scale bar is 1 cm), (**b**) the effect of the actuation on the radius of curvature, (**c**) the actuator’s block force before (peach color) and after (green color) the durability tests (**d**) the electrical response measurements for the 16 electrodes on the actuator before and after 100 actuations.

The durability study of the developed actuator assesses its performance throughout repeated use over 100 actuation cycles, focusing on conformability, force generation, and electrical response. Figure 6a illustrates the actuator’s conformability at 0, 10, 25, 50, and 100 actuations, with Figure 6b quantifying this by plotting the radius of curvature against the number of actuations. This analysis reveals how well the actuator maintains its flexibility over time. Figure 6c compares the actuator’s block force before and after the durability tests, offering insights into the retention of its force-generating capabilities. Notably, Figure 6d showcases the electrical response measurements for all 16 electrodes on the actuator before and after 100 actuations.

The study observes a consistent trend of increasing electrical response from the first to the sixteenth electrode, both before and after repeated actuation. This persistence suggests good electrical durability and consistency in the actuator’s behavior. While specific data points are not provided, the holistic approach of this study, examining multiple aspects of the actuator’s functionality, provides valuable insights into its long-term performance and reliability. Such information is crucial for predicting the actuator’s behavior in practical applications and identifying potential areas for improvement in its design or materials.

#### 3.2.4. Environmental Testing of the Actuator

**Figure 7:**
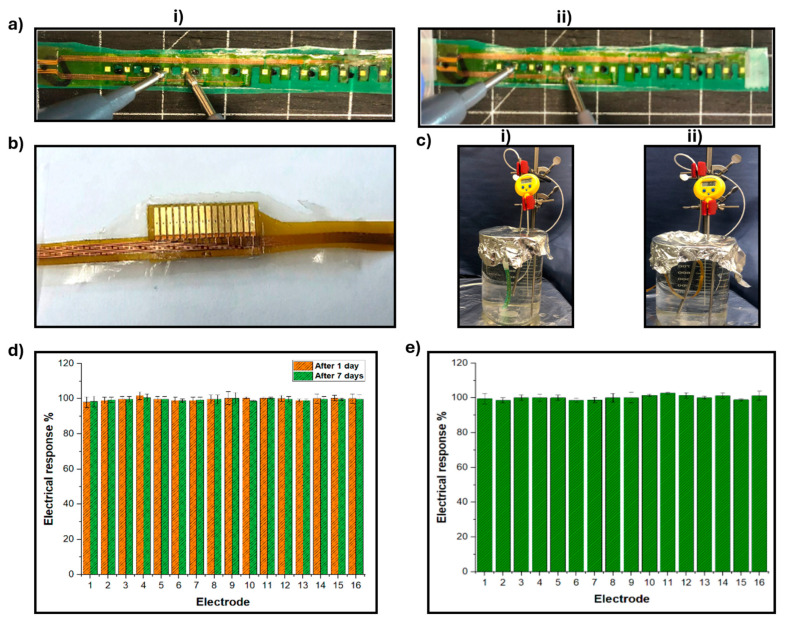
(**a**) Electrical response measurements between (**i**) neighboring and (**ii**) cross electrodes, (**b**) flex PCB/extender junction, (**c**) (**i**) linear actuator immersed in a saline medium under 37 °C for 7 days (**ii**) flex PCB/extender junction immersed in a saline medium under 37 °C for 1 day, (**d**) electrical resistance measurements for the 16 electrodes on the actuators at dry conditions, (after 1 and 7 days in saline at 37 °C, and (**e**) electrical response measurements for the 16 electrodes on flex PCB/extender at dry conditions and after days in saline at 37 °C.

The environmental testing of the actuator, as depicted in Figure 7, provides crucial insights into the performance and durability of the SRSA under conditions mimicking physiological conditions. This series of experiments is essential for evaluating the device’s potential for clinical cardiac applications, focusing on the actuator’s conductivity and the stability of the junction between the flex PCB and extenders.

The study began by examining the electrical response between neighboring and cross-electrodes (Figure 7a), establishing a baseline for the actuator’s electrical performance. This was followed by an investigation of the flex PCB/extender junction (Figure 7b), which is a critical component for signal transmission in the device. A key aspect of the environmental testing involved immersing the linear actuator in a saline medium at 37 °C for an extended period, 7 days for the actuator itself and 1 day for the flex PCB/extender junction (Figure 7c). This setup closely simulates the conditions within a heart chamber, allowing researchers to assess how the device’s electrical properties might change over time in a physiological environment.

The results of these tests are presented in Figure 7d,e. Figure 7d shows the electrical resistance measurements for the 16 electrodes on the actuators under dry conditions and after 1 and 7 days of saline immersion at 37 °C. The stability of these measurements across different time points and conditions demonstrates the robustness of the actuator’s electrical properties in a simulated physiological environment. Figure 7e further elaborates on this by comparing the electrical response measurements for the 16 electrodes on the flex PCB/extender junction under dry conditions and after 1 and 7 days in saline at 37 °C. The consistency of these results indicates that the flex PCB extender junction maintains its electrical integrity even when exposed to conditions mimicking the cardiac environment.

These findings are particularly significant for the clinical application of SRSAs. The stability of the electrical responses in both the actuator and the flex PCB/extender junction under simulated physiological conditions suggests that the device can maintain its functionality and reliability when deployed in a cardiac setting. This durability is crucial for any implantable medical device, especially one designed for the challenging environment of the heart. Moreover, the consistency of results across dry and saline-immersed conditions indicates that the SRSA can provide reliable sensing and actuation capabilities regardless of its surrounding medium. This is a critical factor for its potential use in various cardiac procedures or as a long-term implant. These environmental tests provide strong evidence for the SRSA’s ability to withstand and function in conditions like those found in the heart. The stability of the actuator’s conductivity and the robustness of the flex PCB/extender junction under these conditions are indicators of the device’s potential for successful real-world cardiac applications. These results pave the way for further development and potential clinical trials of the SRSA in cardiac interventions and treatments.

#### 3.2.5. Mechanical Integrity Tests

The experiment on the mechanical integrity of the flex PCB/extenders junction in the SRSA device provides crucial insights into its durability and safety. Using an Instron instrument, we applied increasing loads to test the junction’s breaking point.

**Figure 8:**
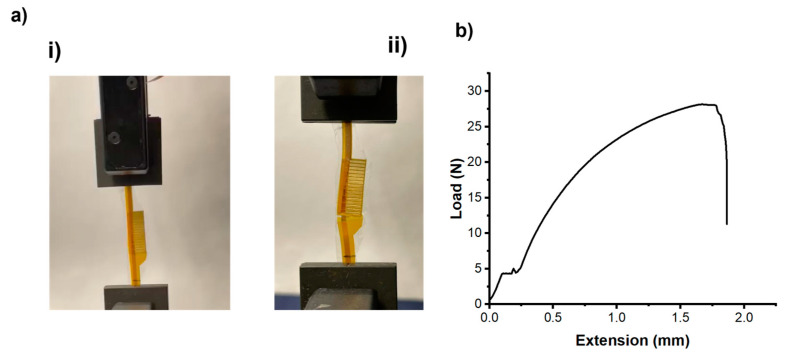
(**a**) (**i**) flex PCB/extender pads before break; (**ii**) flex PCB/extender pads after break. (**b**) Load-extension curve for tensile strength of flex PCB/extender junction pads.

As can be seen in Figure 8, results from three samples showed remarkable consistency in maximum load force, averaging 27.37 N with a minimal standard deviation of ±0.07 N. The maximum extension before failure averaged 1.75 mm, though with higher variability (±1.07 mm). These data demonstrate the junction’s significant strength and manufacturing consistency, crucial for a medical device subject to various stresses during use. The consistent breaking force is valuable for quality control and safety margin calculations, while the variable extension data suggests areas for potential design optimization. These findings are essential for FDA approval, highlighting the device’s mechanical reliability. Future studies could expand on this by including cyclic loading tests, environmental simulations, and failure mode analyses to further enhance the SRSA device’s design and performance in clinical applications.

#### 3.2.6. Medical-Grade Films

The study evaluated the suitability of various medical-grade materials with proven use in FDA-approved devices for their use in SRSAs, focusing on polyurethane-based materials like Pebax, TSP, and Pellethane at different thicknesses (Table S4). The preparation process is well demonstrated, with an example for Pellethane material shown in the Supplemental Materials in Figure S4. Materials underwent heat pressing and block force measurements to assess their performance and suitability. The results in Table 3 show significant variations among the materials. Pellethane 90 AE 1.5 miL demonstrated the highest block force (0.0264 N) and good bonding properties. TSP 1051-5800 1.5 miL also performed well, with consistent results (0.0206 N block force). Pebax variants encountered bonding issues due to higher melting points. The study concluded that TSP 1.5 miL and Pellethane 1.5 miL are optimal for the SRSA device, offering ease of preparation and effectiveness while mimicking the properties of the previously used TPU. These findings provide crucial insights for SRSA development and are a critical part of obtaining compliance with regulatory guidelines for minimally invasive medical devices and highlighting the importance of material properties in SRSA design.

### 3.3. In Vivo Validation of the SRSA Device

The in vivo validation of the SRSA device, as illustrated in Figure 8, demonstrates its potential for cardiac mapping inside the heart chambers of a porcine animal model. This study showcases the device’s ability to be deployed and function effectively in a living heart, marking a significant milestone in its development for clinical applications.

**Figure 9:**
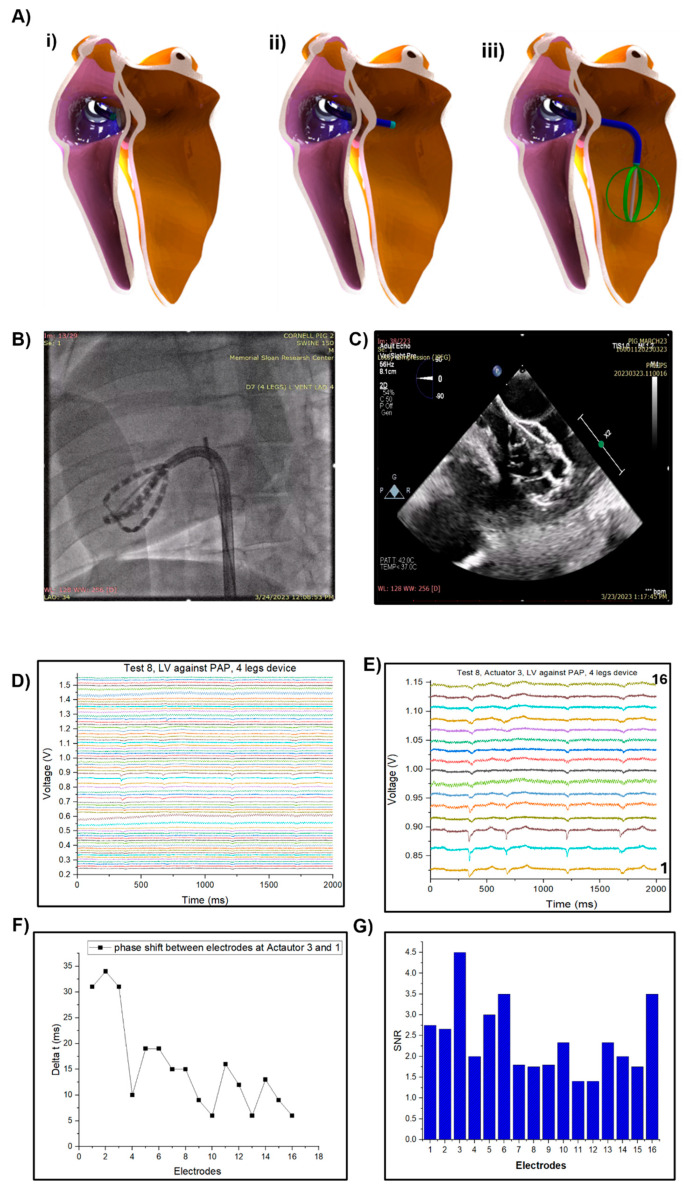
Conformability in cardiac chambers. (**A**) 3D model of catheter delivery progressing from the (**i**) inferior vena cava (IVC), (**ii**) left atrium, and (**iii**) left ventricle. (**B**) Sensor-tissue contact inside the heart location using fluoroscopy after deployment. (**C**) ICE catheter image that shows deployment of SRSA device in LV heart chamber. (**D**) Electrograms acquired while device was in left ventricle. (**E**) Electrograms acquired with one single actuator. (**F**) Phase shift between electrodes at Actuator 1 and 3. (**G**) Signal to noise ratio analysis for acquired electrograms.

Figure 9A provides a series of 3D schematics that demonstrate the access and deployment process of the SRSA device within a left ventricle, the most difficult chamber to access and map (access to the left atrium is obtained by deployment immediately following transseptal puncture; the right heart can be accessed similarly, before transseptal puncture). These models illustrate the progression from the initial state upon catheter insertion (Figure 9A(i)), to catheter entry into the left atrium via a transseptal puncture (Figure 9A(ii)), and finally to the fully deployed state of the device inside the left ventricle (Figure 9A(iii)). This sequence highlights the device’s design for minimally invasive procedures, demonstrating its ability to be inserted and expanded within the confined space of a heart chamber with minimal trauma to the surrounding tissues. The fluoroscopic images in Figure 9B provide visualization of the SRSA device’s deployment inside the heart, demonstrating its ability to conform to the inner surface of the heart chamber by utilizing the soft robotic actuation. This conformability is crucial for ensuring good contact between the device’s sensors and the cardiac tissue, which is essential for accurate signal acquisition. Furthermore, the device did not negatively impact cardiac or valvular function after the procedure, based on echocardiographic images.

Figure 9C displays an ICE catheter image illustrating the SRSA device successfully deployed within the LV chamber. The image clearly shows the device’s conformable positioning along the chamber wall, highlighting the SRSA’s effective placement for optimal contact and signal acquisition.

The SRSA’s functionality is confirmed as presented in Figure 9D, which displays the electrograms as voltage signals collected over time by each of the 64 electrodes from the left ventricle. Figure 9E shows the electrograms acquired by a single actuator in the four-legged device. The presence of clear, distinct electrical signals from all electrodes simultaneously shows the SRSA device’s capability for multi-point sensing. This would allow for a comprehensive mapping of electrical activity across the cardiac tissue, providing a more detailed and nuanced understanding of cardiac electrophysiology than traditional single-point sensing methods.

The ability of all the electrodes to acquire signals simultaneously from the left ventricle, as shown in Figure 9D, underscores the SRSA’s potential for real-time, high-resolution cardiac mapping. This ability is critical for rapidly and efficiently identifying and mapping complex arrhythmias or guiding ablation procedures with high precision. Moreover, the successful deployment and signal acquisition through a minimally invasive procedure highlights the SRSA’s potential to significantly improve cardiac interventions. Providing comprehensive electrical mapping in a shorter time could reduce the required procedural time and reduce procedural risks and recovery times for patients undergoing cardiac diagnostic or therapeutic procedures. This is especially critical for arrythmias like ventricular tachycardia, where clinicians must induce the arrythmia during mapping to optimally resolve it.

The electrical signals obtained from the electrodes were characterized in detail, revealing variable signal quality across the array. The SNR analysis in Figure 9G shows values ranging from approximately 1.4 to 4.5 across the 16 electrodes, with electrode 3 demonstrating the highest SNR (≈4.5) and electrodes 11–12 showing the lowest SNR (≈1.4). With a sampling rate of 1000 Hz, which provides sufficient temporal resolution to capture physiological signals up to 500 Hz according to the Nyquist theorem, the device demonstrated adequate sensing capabilities for cardiac electrical activity. However, the variation in SNR across electrodes suggests potential optimization opportunities in electrode design or placement to achieve more uniform signal quality. The relatively modest SNR values indicate that while the device can detect physiological signals, there may be room for improvement in signal conditioning or the electrode–tissue interface to enhance overall recording fidelity.

The conformability of the device, as evidenced by fluoroscopic images during the minimally invasive procedure, suggests that the SRSA can adapt to the unique anatomical features of the cardiac environment. This adaptability is crucial for ensuring consistent sensor–tissue contact across various locations within the cardiac chamber, potentially improving the accuracy and reliability of cardiac mapping procedures. This in vivo study provides compelling evidence of the SRSA’s applicability for the simultaneous detection of cardiac signals in a living heart model. The device’s ability to be deployed minimally invasively, conform to cardiac structures, and simultaneously acquire clear electrical signals from multiple points within the left ventricle represents a significant advancement in cardiac mapping technology. These results pave the way for further development and potential clinical trials, suggesting that the SRSA could become a valuable tool in the diagnosis and treatment of cardiac conditions, offering clinicians detailed insights into cardiac electrical activity with minimal patient impact.

Despite our results showing promise for various cardiac mapping applications, several issues applications in cardiac mapping, several issues need to be addressed for clinical translation. First and foremost, testing biocompatibility for cytotoxicity, thrombogenicity, and inflammatory response will be conducted to ensure ISO 10993 standards [19]. Another challenge that must be considered is long-term stability across sterilization cycles. Although our environmental testing showed stability over 7 days in various conditions, the device needs to have a shelf life of 6 to 12 months for clinical use. Lastly, scaling the production of our device while optimizing cost-effectiveness is a challenge that warrants careful consideration. Although our current fabrication process is effective for prototype development, automation, and optimization will be vital for mass production.

In comparison to our production cycle, costs will likely tend to increase with the use of medical-grade materials and maintenance of standards for clean room assembly. However, potential reductions in time spent on procedures and improved patient outcomes might offset any rising costs through advanced cardiac mapping. Moving forward, studies will need to assess the device’s capabilities with variations in anatomical geometries and patterns of arrhythmia. Developing standardized protocols for signal processing and mapping will be needed for clinical translation. While the challenges presented may be significant, they embody the standard hurdles of medical device production and validation that will be mitigated as research continues.

## 4. Conclusions

The development and validation of a SRSA for high-resolution cardiac mapping represents a significant advancement in the field of cardiac electrophysiology. This study addresses several limitations of traditional cardiac mapping techniques, such as insufficient electrode coverage, poor conformability to complex cardiac geometries, and high costs. By integrating a 64-electrode array into a soft robotic catheter, the SRSA offers improved contact with cardiac tissue, potentially enabling more accurate and detailed cardiac maps. The dual-layer flex PCB design, combined with a soft robotic actuator, ensures extensive electrode coverage and excellent conformability to the heart’s anatomy. This integration improves the spatiotemporal resolution of the acquired electrical signals, which is crucial for the effective diagnosis and treatment of arrhythmias and can be explored in clinical cases in the future. Unlike some previous methods requiring open-heart surgery, the SRSA can be deployed minimally invasively, reducing patient risk and recovery time. The catheter-based deployment system was rigorously tested, showing successful navigation and deployment within the cardiac chambers.

The SRSA underwent extensive bench testing, including burst/leakage pressure tests, block force measurements, and durability tests, demonstrating high mechanical integrity and resilience. In vivo testing in a porcine model further validated the SRSA’s capability to conform to cardiac anatomy and produce high-resolution electrical maps, confirming its potential for clinical application. The use of flexible and stretchable materials, along with advanced fabrication techniques such as laser-based post-processing, allowed for the creation of a highly conformable and durable SRSA. This innovative approach ensures the device can withstand the dynamic environment of the heart while maintaining optimal performance. These results pave the way for further research and development that should focus on clinical trials to evaluate the SRSA’s performance in human subjects, integration with advanced data analysis tools, exploration of additional biomedical applications, and ongoing optimization and customization of the device. In conclusion, the SRSA represents a transformative advancement in cardiac mapping technology, combining the advantages of soft robotics, stretchable electronics, and minimally invasive procedures. This work not only addresses current limitations in the field but also sets the stage for future innovations that can improve the diagnosis and treatment of cardiac arrhythmias. The integration of this technology into clinical practice holds great promise for enhancing patient care and advancing the field of cardiac electrophysiology.

## Figures and Tables

**Table 1:** Comparison of key features, fabrication methods, and performance characteristics of previously developed soft robotic sensor arrays vs. the present study.

Key Features	Electrode Array	Fabrication	Animal Testing	Advantages	Limitations	Ref.
Soft robotic array with hydraulic actuation	128 electrodes, Cu layer between PI	13.5 Fr catheter, 40–50 μm thick	Porcine cadaver	High conformability (85–90%)	Limited in vivo validation	[6]
Bioresorbable transparent array	16 electrodes, Mo nano grid	PLGA substrate, 1000 nm thick	Rat model	Transparent, bioresorbable	Requires open surgery	[12]
Multilayer sensing/actuation array	64 electrodes (8 × 8), Au on PI	3.3 μm PI layers, customizable catheter	Rabbit and human hearts	Multimodal sensing	Complex fabrication	[15]
Inflatable basket design	Not specified	Eight TPU layers, <50 μm	Cadaveric porcine	8 Fr compatible	Limited sensing integration	[13]
Large-area sensor web	Multiple sensors, ultrathin	<5 μm thick	Rabbit and pig models	Large area coverage	Complex geometry	[16]
4-spline soft robotic array	64 electrodes (4 × 16), ENIG-coated Cu	13.8 Fr catheter, medical-grade materials	Live porcine model	Complete in vivo validation, simplified fabrication	Cost considerations	Present study

**Table 2:** A summary of the key quantitative and qualitative data for the different tested samples.

Sample	Failure Mode	Maximum Pressure (kPa)	Maximum Volume (mL)	Volume at Burst (mL)
Actuator sub-assembly (four-legged extension)	Elastomer extension Pleat windows detachment Leakage	186	6.89	12
Actuator sub-assembly (single-leg extension)	Elastomer extension in a single leg Leakage	172	5.43	7.18
Sensing actuator	Leakage Elastomer extension in a single leg	186	5.32	10.3
4.85 mm PVA width actuator sub-assembly	No leakage observed The pressure was reduced while the elastomer kept inflating/extending in two legs until it burst.	186	7.29	11.20
4.35 mm PVA width actuator sub-assembly	No leakage observed The pressure was reduced while the elastomer in the first leg kept inflating/extending. Then again, the pressure increases while at the same failure mode starts occurring in the 2nd and 3rd legs. The burst failure mode happened in leg 1.	220	6.55	8
3.85 mm PVA width actuator sub-assembly	Leakage observed at one of the legs as well as at the device inlet location The pressure was reduced while the actuator was leaking.	35	7	N/A

**Table 3:** Different medical-grade materials used to prepare actuators and their block force values.

Medical-Grade Material	Actuator Fabrication Conditions	Block Force Value	Comments
Pellethane 2363 80 AE 1 miL	5 min heat press at 137 °C—good bonding between PVA and Pellethane 2363 films	0.0196 N	Heat press linear flex PCB again for 5 min before actuation
Pellethane 90 AE 1.5 miL	10 min heat press at 137 °C—good bonding between PVA and Pellethane 2363 films	0.0264 N	Heat press linear flex PCB again for 5 min before actuation; ideal material
Pellethane 90 AE 2.5 miL	5 min heat press at 137 °C—good bonding between PVA and Pellethane 90 AE	0.0255 N	Heat press linear flex PCB again for 10 min before actuation—challenges with PVA windows
TSP 1065-4500 0.5 miL	10 min heat press at 137 °C—good bonding between PVA and TSP 1051 films	0.0206 N	Difficult assembly given six layers on top of one another; do not recommend 0.5 miL
TSP 1051-5800 1.5 miL	10 min heat press at 137 °C—good bonding between PVA and TSP 1051 films	0.0206 N	Heat press linear flex PCB again for 5 min before actuation; ideal material
TSP 1031-3400 70A 3 miL	10 min heat press at 137 °C—good bonding between PVA and TSP 1051 films	N/A	Heat press linear flex PCB again for 5 min before actuation; do not use tape for heat press; use glass instead
Pebax 7233 SA 01 MED 1 miL	10 min heat press at 149 °C—bonding does not suffice	N/A	Heat press linear flex PCB again for 5 min before actuation; the material’s melting point is 159 °C
Pebax 5533 SA01 2 miL	10 min heat press at 148 °C—bonding does not suffice	0.017 N	Heat press linear flex PCB again for 5 min before actuation; the material’s melting point is 159 °C

## Data Availability

The original contributions presented in the study are included in the article, further inquiries can be directed to the corresponding author.

## Supplementary Materials

The following supporting information can be downloaded at: https://www.mdpi.com/article/10.3390/mi15111393/s1, Figure S1: General overview of the SRSA device color-coded different assembly levels starting with device components, device minor sub-assemblies, and device major sub-assemblies as outlined in Tables S1–S3; Figure S2: Assembled 4-legged SRSA device; Figure S3: (a) Actuator Sub-assembly Fabrication Steps, (b) Schematic Illustration of Burst/Leakage Test Setup, (c) Experimental Setup, (d) Pressure vs Volume Plot for Burst/Leakage Actuator Sub-assembly Validation Test, (e) Sensing Actuator Fabrication Steps and Sample Mounting for Burst/Leakage Tests, (f) Pressure vs Volume Plot for Sensing Actuator Sample, (g) CAD Design of Various PVA Sacrificial-layer Skeleton Width, (h) Assembled Actuators of Various PVA Sacrificial-layer Skelton Width, (i) Volume Plot for Actuator Samples of Various PVA Sacrificial-layer Skeleton Width; Figure S4: Medical grade actuator fabrication protocol and block force measurements setup; Table S1: SRSA Device components and their description; Table S2: SRSA Device Minor Sub-Assemblies Table S3: Device Major Sub-Assemblies; Table S4: Medical grade polyurethane materials tested purchased from Polyzen company.
