# Supplementary material for: Design, Testing, and Validation of a Soft Robotic Sensor Array Integrated with Flexible Electronics for Mapping Cardiac Arrhythmias
Source: Micromachines (Basel). 2024 Nov 18;15(11):1393. doi: 10.3390/mi15111393 (PMC11596174; doi:10.3390/mi15111393)
Supplement: Supplementary file 1 [file micromachines-15-01393-s001.zip › micromachines-3269432-supplementary.pdf]

Design, Testing, and Validation of a Soft Robotic Sensor Array Integrated with Flexible Electronics for Mapping Cardiac Arrhythmias

Abdellatif Ait Lahcen[1], Michael Labib[1], Alexandre Caprio[1], Mohsen Annabestani[1], Lina Sanchez-Botero[1], Weihow Hsue[2], Christopher F Liu[3], Simon Dunham[1*], Bobak Mosadegh[1*]

[1]Dalio Institute for Cardiovascular Imaging, Department of Radiology, Weill Cornell Medicine, New York, NY 10021, USA

[2]Department of Clinical Sciences, College of Veterinary Medicine, Cornell University, Ithaca, NY 14853, USA
[3]Department of Cardiology, Weill Cornell Medicine, New York, NY 10021, USA

*Authors to whom correspondence should be addressed: sid2012@med.cornell.edu ; bom2008@med.cornell.edu

Supplementary Materials

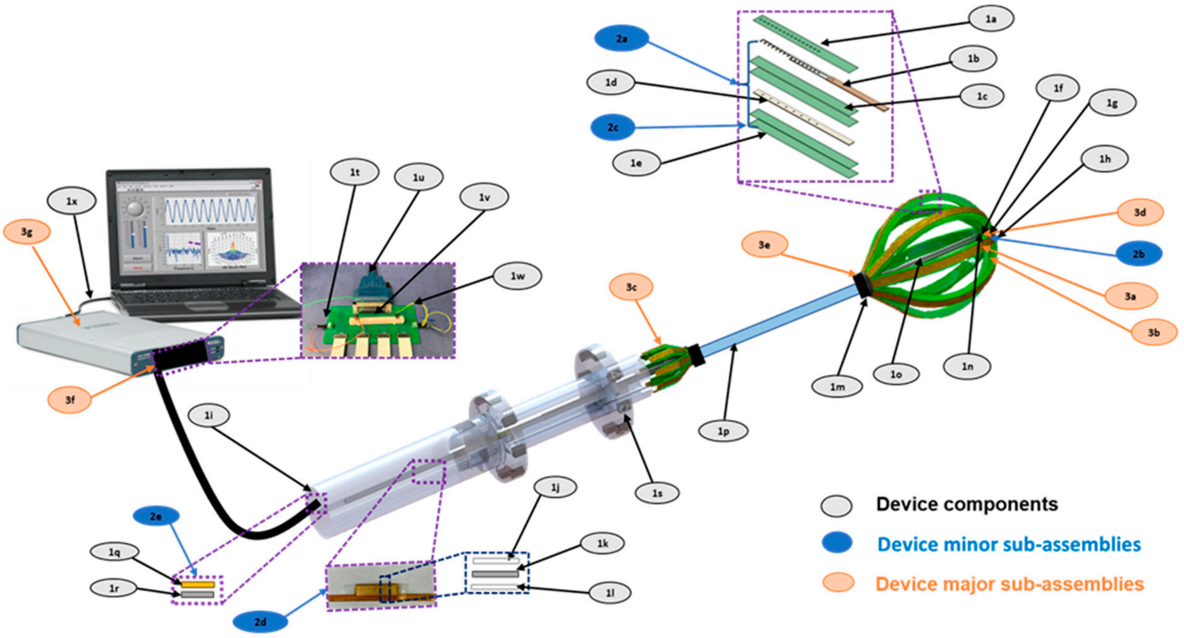

Figure S1: General overview of the SRSA device color-coded different assembly levels starting with device components, device minor sub-assemblies, and device major sub-assemblies as outlined in **Tables S1**, **S2**, and **S3**.

Table S1: SRSA Device components and their description

Device components	Description
**1a- Sensor insulation**	A- 1-layer of green thermal polyurethane (TPU) laser cut windows that is used to insulate the flex PCB top layer.
**1b- Sensor array**	4 flex PCBs that are used to assemble the 4-legged sensor array.
**1c- Actuator upper elastomer layer**	A 2-layer GPU sheet is used on the top of the PVA sacrificial layer. The edges of the sheet are cut off and divided into enough parts to cover the balloon skeleton.
**1d- Actuator sacrificial Layer**	This component is used as a sacrificial layer and is sandwiched in between the top actuator TPU and bottom actuator TPU.
**1e- Actuator lower elastomer layer**	A 2-layer GPU is used on the bottom of the PVA sacrificial layer. The edges of the sheet will be cut off and will be divided into enough parts to cover the balloon skeleton.
**1f- Distal Hub interface upper elastomer**	A 2-layer GPU sheet that is heat-pressed on the top of the PTFE inlet.
**1g- Distal Hub bonding prevention layer**	This intermediate sub-assembly tool is used to prevent the actuator inlet from bound with the 2 layers of TPU
**1h- Distal Hub interface lower sealing TPU**	A 2-layer green TPU sheet will be heat-pressed on the bottom of the PTFE inlet.
**1i- Sensor array electrical extenders**	This component is used to extend the length of the leg sensor array
**1j- Extended electrical interface top insulation**	1-layer transparent TPU that will be used to mechanically seal the top of the PADs junction between Flex PCB and the extender.
**1l- Extended electrical interface bottom insulation**	1-layer transparent TPU that is used to mechanically seal the bottom of the PADs junction between Flex PCB and the extender.

**1m- Proximal actuator attachment heat shrinks**	The PVC heat shrink tubing will be used to attach the sensing actuator assembly to the outer rod and it is reinforced using glue.
**1n- Distal Hub hydraulic tape**	PTFE tape is used to wrap the balloon inlet to the inner rod at the distal hub location.
**1o- Inner rod**	This component is used to connect to the balloon actuator inlet at the distal hub location.
**1p- Outer rod**	This component is used to as a support to attach each leg sensor array at the proximal hub using a heat shrink and glue to hold it together.
**1q- Flex-to-Rigid interface**	This component will be used to connect the sensing actuator to the ZIF connector in the rigid PCB.
**1r- ZIF flat PADs junction Z-tape**	This component is used to connect the flex PCB extenders and the long ZIF flat connector.
**1s- 3D-printed manifold**	This intermediate sub-assembly tool is used to align the 4-legged sensors array
**1t- Rigid PCB**	This component is used to connect the 4-legged sensor arrays to the DAQ.
**1u- National Instrument (NI) Cable**	This component is used to connect the NI DAQ to the rigid PCB D-sub pinouts.
**1v- Breadboard**	This component is used to connect the ground pins.
**1w- Jumper wires**	This component will be used to connect the ground pins in the rigid PCB to the breadboard.
**1x- USB-B type**	This component is used to connect the NI DAQ to the PC.

Table S2: SRSA Device Minor Sub-Assemblies

Device minor sub-assemblies	Description
**2a-** Actuator upper elastomer layer/Actuator sacrificial layer	This sub-assembly contains the laser cut actuator sacrificial layer heat pressed on the 2 layers of the actuator upper elastomer layer.
**2b-** Distal hub bonding prevention layer sandwiched between distal hub interface upper and lower TPU	The laser cut bonding prevention layer is sandwiched between 2 layers distal hub interface upper and lower elastomers (PTFE inlet top sealing TPU and PTFE inlet bottom sealing TPU). This inlet will serve to connect the inner rod to the actuator sub-assembly.
**2c-** Actuator lower elastomer (TPU) layer punctured hole	2-layer TPU in the bottom actuator TPU with a punctured hole in the middle using a biopsy punch. This punctured hole serves to combine the bottom actuator TPU and the PTFE inlet.
**2d.** Insulated and shortened sensor array with extended electrical interface	This sub-assembly contains the insulated and shortened sensor array, the interface z-axis conductive tape, and the extended electrical interface top/bottom insulation.
**2e.** ZIF flat connector/flex PCB extenders junction	This sub-assembly contains the flex-to-rigid interface and the ZIF flat PADs junction z-tape.

Table S3: Device Major Sub-Assemblies

Device major sub-assemblies	Description
**3a- Sensing actuator sub-assembly**	This sub-assembly contains the **4-legged sensor arrays** and the **actuator sub-assembly**.
**3b- Actuator sub-assembly**	This sub-assembly contains the distal hub bonding prevention layer sandwiched between the distal hub upper and lower elastomer (TPU), the actuator lower elastomer layer punctured hole, and the actuator upper elastomer/actuator sacrificial layer. This sub-assembly serves for device actuation.
**3c- Leg sensor array**	This sub-assembly will contain the **Insulated and shortened sensor array with extended electrical interface** and **ZIF flat connector/flex PCB extenders junction.**
**3d- Distal-hub attachment sub-assembly**	This sub-assembly contains the **inner rod** that is inserted inside the Distal hub bonding prevention layer sandwiched between the distal hub interface upper and lower TPU of the sensing actuator sub-assembly and wrapped with PTFE tape.
**3e- Proximal hub assembly**	This assembly contains the **Distal-hub** attachment sub-assembly, and the proximal actuator attachment heat shrink used to attach the **4-legged sensor arrays** to the **outer rod**.
**3f- Rigid PCB sub-assembly**	This sub-assembly contains the rigid PCB, the breadboard, and the jumper wires.
**3g- Data acquisitor (DAQ)**	This sub-assembly contains the Data acquisitor (DAQ), the National instruments cable, and the USB-B type.

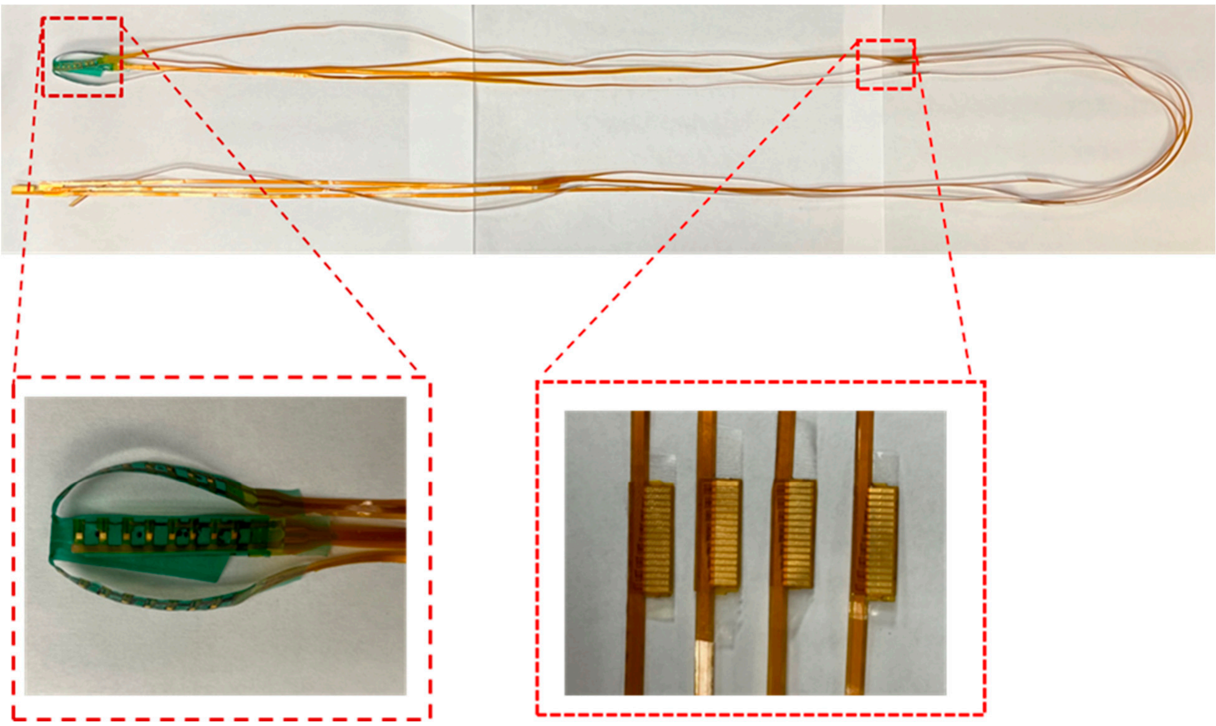

Figure S2: Assembled 4-legged SRSA device

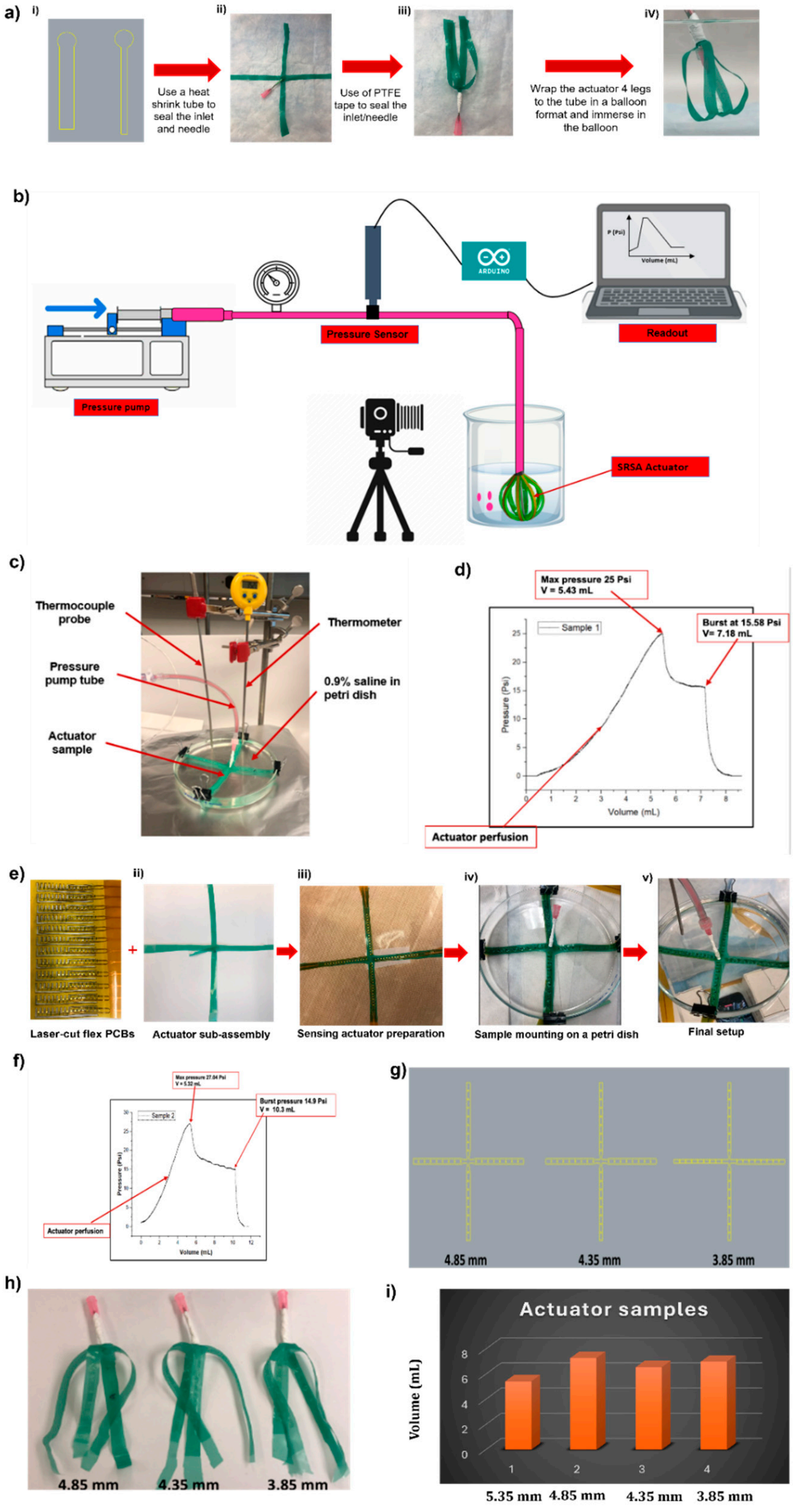

Figure S3. **a)** Actuator Sub-assembly Fabrication Steps, **b)** Schematic Illustration of Burst/Leakage Test Setup, **c)** Experimental Setup, **d)** Pressure vs Volume Plot for Burst/Leakage Actuator Sub-assembly

Validation Test, **e)** Sensing Actuator Fabrication Steps and Sample Mounting for Burst/Leakage Tests, **f)** Pressure vs Volume Plot for Sensing Actuator Sample, **g)** CAD Design of Various PVA Sacrificial-layer Skeleton Width, **h)** Assembled Actuators of Various PVA Sacrificial-layer Skelton Width, **i)** Volume Plot for Actuator Samples of Various PVA Sacrificial-layer Skeleton Width.

Table S4: Medical grade polyurethane materials tested purchased from Polyzen company.

SIP1-700	Pellethane 2363 80AE	1mil	Natural	Pellethane 2363 80AE 1mil film on blue polypro carrier
SIP1-701	Pebax 5533 SA01 Med	2mil	matte	Pebax 5533 SA01 Med 2mil matte film
SIP1-702	TSP 1031-3400 70A	3mil	matte	TSP 1031-3400 70A 3mil on blue polypro carrier
SIP1-703	Pellethane 90AE	1.5mil	matte	Pellethane 90AE 1.5mil on blue PP carrier
SIP1-704	Pellethane 90AE	2.5mil	matte	Pellethane 90AE 2.5mil on blue PP carrier
SIP1-705	Pebax 7233 SA01 Med	1mil	matte	Pebax 7233 SA01 Med
SIP1-706	TSP 1051-5800	1.5mil	matte	TSP 1051-5800 1.5mil
SIP1-707	TSP 1065-4500 on paper	0.5mil	matte	TSP 1065-4500 0.5mil film on paper

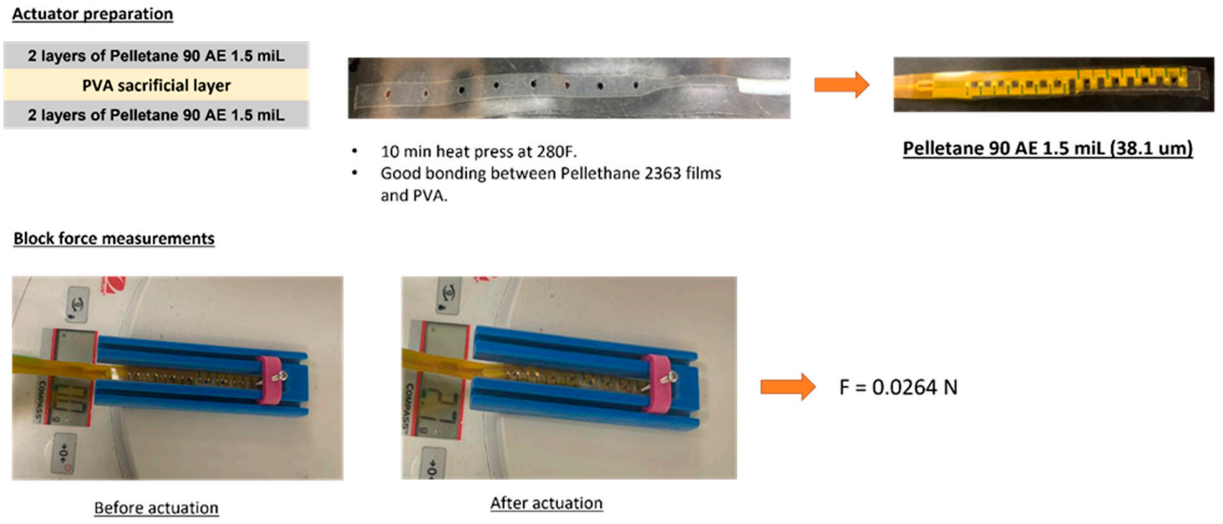

Figure S4. medical grade actuator fabrication protocol and block force measurements setup
